# Plasma levels of leptin and mammographic density among postmenopausal women: a cross-sectional study

**DOI:** 10.1186/bcr1603

**Published:** 2006-09-29

**Authors:** Anne Stuedal, Giske Ursin, Marit B Veierød, Yngve Bremnes, Janne E Reseland, Christian A Drevon, Inger T Gram

**Affiliations:** 1 Department of Nutrition, University of Oslo, Norway; 2 Department of Preventive Medicine, University of Southern California, Los Angeles, California, USA; 3 Department of Biostatistics, University of Oslo, Norway; 4 Department of Preventive Medicine, Institute of Community Medicine, University of Tromsø, Norway; 5 Department of Biomaterials, Faculty of Dentistry, University of Oslo, Norway

## Abstract

**Introduction:**

Obesity has been linked to increased risk of breast cancer in postmenopausal women. Increased peripheral production of estrogens has been regarded as the main cause for this association, but other features of increased body fat mass may also play a part. Leptin is a protein produced mainly by adipose tissue and may represent a growth factor in cancer. We examined the association between leptin plasma levels and mammographic density, a biomarker for breast cancer risk.

**Methods:**

We included data from postmenopausal women aged 55 and older, who participated in a cross-sectional mammography study in Tromsø, Norway. Mammograms, plasma leptin measurements as well as information on anthropometric and hormonal/reproductive factors were available from 967 women. We assessed mammographic density using a previously validated computer-assisted method. Multiple linear regression analysis was applied to investigate the association between mammographic density and quartiles of plasma leptin concentration. Because we hypothesized that the effect of leptin on mammographic density could vary depending on the amount of nondense or fat tissue in the breast, we also performed analyses on plasma leptin levels and mammographic density within tertiles of mammographic nondense area.

**Results:**

After adjusting for age, postmenopausal hormone use, number of full-term pregnancies and age of first birth, there was an inverse association between leptin and absolute mammographic density (*P*_trend _= 0.001). When we additionally adjusted for body mass index and mammographic nondense area, no statistically significant association between plasma leptin and mammographic density was found (*P*_trend _= 0.16). Stratified analyses suggested that the association between plasma leptin and mammographic density could differ with the amount of nondense area of the mammogram, with the strongest association between leptin and mammographic absolute density in the stratum with the medium breast fat content (*P*_trend _= 0.003, *P *for interaction = 0.05).

**Conclusion:**

We found no overall consistent association between the plasma concentration of leptin and absolute mammographic density. Although weak, there was some suggestion that the association between leptin and mammographic density could differ with the amount of fat tissue in the breast.

## Introduction

Obesity has been associated with increased risk of postmenopausal breast cancer in epidemiological studies [1-3]. The increased conversion of androgens to estrogens by the aromatase enzyme in peripheral adipose tissues [4] along with reduced levels of serum sex hormone binding globulin have been hypothesized to be the main link between obesity and increased risk of postmenopausal breast cancer [2].

Whether the influence of estrogens on the breast tissue is direct or is mediated via other factors, however, has not been established [5]. In addition to being the main site for production of postmenopausal estrogens, adipocytes secrete a number of biological active polypeptides, the adipocytokines [6], some of which may be involved in breast cancer development [7].

Leptin, the most thoroughly studied adipocytokine, is a protein hormone produced mainly by the white adipose tissue [8,9], but is also expressed at other sites, such as in the gastric epithelium [10], the placenta [11], osteoblasts [12], skeletal muscle cells [13] and mammary epithelium [14]. Leptin regulates appetite and energy expenditure by signaling nutritional status to the hypothalamus [15], but is also involved in a number of other processes including the regulation of reproduction and immune response [16,17].

Leptin may act as a growth factor in cancer [18], including the epithelial cancers of colon and breast [19]. Leptin promotes angiogenesis [20-22] and might thereby directly stimulate growth of breast cancer cells [23].

The radiographic appearance of a mammogram is determined by the relative amounts of translucent fat tissue to the denser epithelial and stromal (fibrous) tissues [24]. Mammographic density is a measure of the radiodense area on the mammogram. Both the amount of radiodense tissue (absolute mammographic density) and the percentage of total breast area that appears radiologically dense (percentage mammographic density) have been shown to be associated with breast cancer risk; women with the most dense mammograms having a four to six times higher risk of developing breast cancer compared with women with no densities [25-27]. It was recently shown that ductal carcinoma *in situ *tumors tend to arise in the area of the breast corresponding to the dense part of the mammogram [28], and it has been suggested that mammographic density represents an early biomarker for breast cancer risk [29].

In the present cross-sectional study, we wanted to examine the association between plasma leptin concentration and mammographic density. We hypothesized that the growth-promoting properties of leptin, by stimulating proliferation of epithelial tissue and/or stromal tissue of the breast, could potentially increase the density of the mammogram. As absolute mammographic density is under smaller influence by breast fat and body fat measures than percentage mammographic density, we used absolute density as the main mammographic density variable in our analyses.

## Materials and methods

### Study population

We used data from the Tromsø Mammography and Breast Cancer Study, which aims to identify genetic, hormonal, reproductive and lifestyle characteristics associated with mammographic patterns/densities that may enhance the risk of developing breast cancer [30]. Briefly, women aged 55 and older, residing in the municipality of Tromsø, who attended the National Breast Cancer Screening Program at the University Hospital of North Norway during spring 2001 and 2002, were invited to participate in the study. A total of 1,041 women agreed to participate. This accounted for 70.2% of the women attending the breast cancer screening program during recruitment. Of the 1,041 women, we excluded 22 women with new or previous breast cancer and one woman currently using chemotherapy.

A blood sample was drawn and anthropometric measures were obtained from the participants on the day of the mammographic screening. The study subjects were interviewed by a trained nurse about their current and previous postmenopausal hormone therapy use, their reproductive and menstrual factors, their previous history of cancer and their smoking status. The participants were asked to complete either a four-page questionnaire (2001 participants) or an eight-page questionnaire (2002 participants) at home. The questionnaires contained items on demographic, menstrual and reproductive factors, as well as lifestyle and dietary factors. The study was approved by the Regional Committee for Medical Research Ethics and the National Data Inspection Board. All participants gave written informed consent.

### Assay of plasma samples

Nonfasting venous samples were obtained from the participants on the day of mammographic screening. Samples were stored at -20°C or colder until analysis in December 2002. Samples had been thawed once during storage. The plasma leptin concentration was measured by a competitive radioimmunoassay (Linco Research, St Charles, MO, USA) with recombinant ^125^I-leptin as a tracer [31]. The intra-assay coefficient of variation was 2.4%, whereas the inter-assay coefficient of variation was 6.6%. Leptin measurements were available for 975 women.

### Processing of mammograms

Absolute and percentage mammographic densities were determined using the University of Southern California Madena computer-based threshold method of assessing density, a method that has been described and validated elsewhere [32]. Briefly, the cranio-caudal mammographic images are digitized using a high-resolution Cobrascan CX-812 scanner (Radiographic Digital Imaging, Torrance, CA, USA) and were then viewed on a computer screen. The computer software program assigns a pixel value of 0 to the darkest (black) shade in the image and a value of 255 to the lightest (white) shade with shades of gray assigned intermediate values.

A reader first defines the total breast area using a special outlining tool. Next, the region of interest, excluding the pectoralis muscle, prominent veins and fibrous strands, is defined. The reader then uses a tinting tool to apply a yellow tint to dense pixels with grey levels at or above some threshold *X *and a pixel value of 255. The reader searches for the best threshold where all pixels *X *within the region of interest are considered to represent mammographic densities. The software estimates the total number of pixels and the number of tinted pixels within the region of interest.

The absolute density represents the count of the tinted pixels within the region of interest. The percentage density, or the fraction (%) of the breast with densities, is the ratio of absolute density to the total breast area. As a measure of breast adipose tissue, we used the mammographic nondense area, which we estimated as the total breast area minus the absolute density. All measurements were made on the mammogram from the left breast. The density assessments were performed by GU, whereas the breast area measurements were conducted by a research assistant trained by GU. The readers were blinded to all subject characteristics.

### Data analysis

In our preliminary analyses, we used analysis of variance to study the associations between leptin and selected variables, and the associations between absolute mammographic density and the same selected variables. We also conducted these analyses adjusted for body mass index (BMI).

The association between plasma leptin concentration and mammographic density was studied by multiple linear regression analysis with mammographic density as the outcome variable. Consistent with our previously reported findings on percentage mammographic density from this study [30], we found that absolute mammographic density decreased with higher BMI, with increasing number of full-term pregnancies and with lower age at first birth. We adjusted for these variables in the multivariate analyses, in addition to age, current use of postmenopausal hormone therapy and mammographic nondense area. In the multivariate analyses, leptin was categorized into quartiles, and the covariates were modeled as categorical variables with the following categories: BMI (<20, 20–22, 23–24, 25–26, 27–28, 29–31, 32–34, >34), age (tertiles), number of full-term pregnancies (0, 1–2, 3, >3), age at first birth (<20 years, 20–24 years, >24 years), current postmenopausal hormone therapy use (yes/no) and breast fat tissue (tertiles). In the stratified analyses, BMI was categorized as <23, 23–24, 25–26, 27–28, >28.

To meet the assumptions of normality of residuals from the regression analyses, both the leptin concentration and mammographic density were log_10_-transformed in the analyses where they represented the outcome variable. Back-transformed means and 95% confidence intervals are presented. Test for trends across categories of variables were performed by treating the categories as continuous variables in the analyses. For the multivariate analyses on leptin concentration and mammographic density, complete information was available for 967 women.

Leptin concentration was correlated with the body fat measures of BMI (Spearman's rank correlation, *r*_sp _= 0.56, *P *< 0.001), waist circumference (*r*_sp _= 0.52, *P *< 0.001) and breast fat tissue (*r*_sp _= 0.40, *P *< 0.001). The body fat measures of BMI, breast fat tissue and waist circumference were also correlated (0.66 ≤ *r*_sp _≤ 0.87, with the highest correlation of 0.87 between BMI and waist circumference). Because we wanted to investigate the effect of leptin as a possible growth factor independent of body fat, we performed several analyses adjusting for BMI, mammographic nondense area (representing breast fat) and waist circumference one at a time or together, and with various categorizations of the variables.

In the following, we present results adjusted for BMI and/or mammographic nondense area. Additional adjustment for waist circumference, smoking and alcohol consumption yielded essentially similar results, and are not presented.

Furthermore, because of the correlation (*r*_sp _= 0.56) between our main independent variable, leptin concentration, and BMI, we additionally performed our multivariate analyses with leptin concentration adjusted for BMI by the residual method [33].

We hypothesized that the association between plasma leptin concentration and absolute mammographic density could be modified by the amount of fat tissue in the breast. We therefore also performed multivariate analyses within tertiles of the mammographic nondense area.

Of the 967 women in the analyses, 898 reported one or more full-term pregnancies. For 82 of these women, information on age at first birth was not available. In order to keep these women in the adjusted analyses we replaced the missing values with the median age at first birth for women with the same age and same number of full-term pregnancies.

From the multiple linear regression model of plasma leptin and absolute mammographic density, we report *R*^2 ^values for the total model and the *R*^2 ^change values for the plasma leptin concentration. The statistical analyses were performed using SPSS for Windows (version 11.0; SPSS Inc., Chicago, IL, USA). All *P *values are two-sided. We considered *P *< 0.05 statistically significant.

## Results

The characteristics of the study subjects are summarized in Table 1. The median absolute mammographic density was 14.7 cm^2 ^(range, 0–155.2 cm^2^) and the median plasma leptin concentration was 14.5 ng/ml (range, 1.0–72.0 ng/ml).

Table 2 presents the associations between selected variables and leptin concentration as well as absolute mammographic density. After adjustment for BMI, the leptin concentration decreased statistically nonsignificantly with higher age at first birth, and smokers had significantly lower plasma leptin levels than nonsmokers. Moreover, plasma leptin concentration was positively and statistically significantly associated with BMI, waist circumference and the nondense area of the mammogram. Absolute mammographic density decreased statistically significantly with age, with increasing number of full-term pregnancies and was lower with an early first birth. Women using postmenopausal hormone therapy had a significantly higher absolute mammographic density, on average 5.3 cm^2^, compared with non-hormone users. Absolute mammographic density was inversely and statistically significantly associated with BMI, waist circumference and the nondense area of the mammogram.

### Plasma leptin concentration and mammographic density

Plasma leptin levels and absolute mammographic density were weakly correlated (*r*_sp _= -0.12, *P *< 0.001), and a statistically significant inverse association was found in the unadjusted regression analysis (Table 3). After adjustment for age, number of full-term pregnancies, age at first birth, use of postmenopausal hormone therapy, BMI and mammographic nondense area, this inverse association was no longer apparent. The pattern was essentially the same for the percentage mammographic density (Table 3). When we replaced the leptin concentration with that adjusted for BMI by the residual method in the multivariate analyses, the association between leptin concentration and mammographic density remained essentially unchanged. For the absolute density *P*_trend _changed from 0.32 to 0.28, and for the percentage density *P*_trend _changed from 0.58 to 0.43 (analyses not adjusted for nondense mammographic area) (results not shown).

In analysis stratified by tertiles of mammographic nondense area, no statistically significant association was found between the plasma leptin concentration and the absolute mammographic density in the strata representing the women with the lowest or the highest breast fat content (Table 4). In the stratum with the medium nondense area, the absolute mammographic density increased statistically significantly across quartiles of leptin. Women in the highest quartile of plasma leptin concentration had, on average, 9.7 cm^2 ^higher absolute mammographic density compared with the women in the lowest quartile (*P*_trend _= 0.003). This apparent effect modification was statistically borderline significant (*P *for interaction = 0.05). Stratified analyses not adjusted for BMI gave essentially the same results (*P *for interaction = 0.06). For the percentage density, the association between leptin concentration and mammographic density resembled those of the absolute mammographic density, with an inverse association in the lower stratum of nondense mammographic area (*P*_trend _= 0.05), a positive association in the medium stratum (*P*_trend _= 0.004) and no association in the upper stratum (*P*_trend _= 0.76) (results not shown).

We also performed the analysis on leptin concentration and absolute mammographic density in the strata of the second tertile and the third tertile of nondense mammographic area combined. In this new stratum, the absolute density was on average 3.9 cm^2 ^higher in the highest quartile of plasma leptin concentration compared with the lowest quartile (*P*_trend _= 0.12, *P *for interaction = 0.23) (results not shown). When we restricted the analysis to women having a BMI between 18 and 40 (*n *= 945) or to women in the 5th-95th percentiles of nondense mammographic area (*n *= 872), or to non-current hormone therapy users (*n *= 714), the associations between leptin concentration and mammographic density were essentially unchanged.

In the adjusted analyses of plasma leptin levels and absolute mammographic density, with a model where both BMI and nondense area were included, the *R*^2 ^value was 22.6% (*P *< 0.001) and the *R*^2 ^change value for leptin concentration was 0.6% (*P *= 0.05).

## Discussion

Epidemiological studies on leptin and breast cancer have been scarce and inconclusive. One small case-control study found no association between the serum leptin concentration and breast cancer among postmenopausal women, but found an inverse association among premenopausal women [34]. Another small case-control study performed among premenopausal women [35] reported a statistically nonsignificant positive association between serum leptin levels and the risk of carcinoma *in situ *of the breast. A Swedish nested case-control study on postmenopausal women [36] found no statistically significant association between plasma levels of leptin and breast cancer risk. One earlier study [37] evaluated the association between circulating leptin levels among premenopausal women and mammographic density, assessed by the Wolfe's classification system. In that study, leptin was inversely associated with high-risk mammographic patterns. This is in accordance with our analyses not adjusted for body fat measures.

The present study found a crude inverse association between leptin concentration and mammographic density. This association disappeared after adjustment for body fat measures. The small *R*^2 ^change of 0.6% for leptin concentration suggests a marginal, if any, role for leptin in determining mammographic density. We did find, however, a statistically significant increase in mammographic density across quartiles of leptin concentration among the women with a medium content of nondense tissue or breast fat.

Because leptin is produced and secreted mainly from the adipose tissue, it may seem inappropriate from a biologic point of view to separate the association between leptin concentration and mammographic density from that of the association between body fat and mammographic density. Furthermore, it may seem puzzling that fat measures are inversely associated with mammographic density, whereas tentatively a positive association could exist between leptin and mammographic density, as suggested from our stratified analyses adjusted for fat measures.

Obesity is associated with increased breast cancer risk in postmenopausal women [1]. Paradoxically, obesity has shown to be associated with favorable mammographic patterns, regardless of methods of mammographic assessment [38-40]. The reason why obesity should be related to a smaller amount of fibroglandular tissue in the breast is unclear [41]. To our knowledge it is not known what determines the relative amounts of fat and stromal tissue during human female breast development. Interestingly, it is well known from dairy science that high-energy feeding in heifers (a young cow that has not had a calf) increases mammary fat deposits and decreases the number of epithelial cells [42]. It has been suggested that leptin might, via a regulatory effect on DNA synthesis in bovine mammary epithelial cells, mediate such an effect [43]. Furthermore, even if body fat measures are inversely associated with mammographic density, there could still be positive associations between specific fat-related substances [6] and mammographic density. To investigate an association between leptin and mammographic density, we therefore had to adjust for the confounding effect of body fat.

Although percentage mammographic density is a more commonly used estimate, absolute density has also been associated with breast cancer [26,27,44,45]. Absolute density may be a more appropriate marker in studies where body fat mass is investigated, such as in dietary studies [46-48].

A role for leptin in breast cancer tumorigenesis has been hypothesized based on the detection of leptin protein in human breast tumors [49], the detection of leptin receptors and the proliferative effect of leptin in breast cancer cell lines [23,50,51]. Lack of oncogene-induced mammary tumors in genetically obese mice, leptin-deficient (ob/ob) mice [52] or leptin-receptor-deficient (db/db) mice [53] have also been discussed in light of this hypothesis. The influence of leptin on normal breast tissue, however, is less investigated. Leptin receptors have been detected in the normal human breast epithelial HBL100 cell line [23], whereas investigators failed to demonstrate any significant immunoreactiviety of the leptin receptor (OB-R) in normal breast gland [54]. Leptin mRNA expression has been detected in normal breast tissue and in secretory breast epithelial cells [14], in benign epithelial hyperplasia as well as in normal epithelial cells in the vicinity of malign lesions [55]. Breast epithelial cells, therefore, might under certain circumstances themselves produce and/secrete leptin. Normal breast tissue appears to have lower or absent leptin expression compared with cells of benign breast lesions and malignant ductal cells [54,55]. These observations could indicate a role for leptin during malignant transformation of breast cells.

Although it is particularly the effect of leptin on epithelial mammary duct cells of the breast that has been studied, leptin might promote growth in other cell populations of the breast, such as fibroblasts [56], and thereby cause stromal alterations reflected by increased mammographic density [57].

Because the level of leptin in breast fat might be biologically important, it seems probable that plasma levels as a marker for breast level leptin are only comparable in women with the same amount of breast adipose tissue – or, in other words, that breast adipose tissue may be a modifier of the leptin-mammographic density association. We therefore stratified our analyses on the mammographic nondense area, and a role for breast fat in modifying the association between leptin and mammographic density was suggested (*P *for interaction = 0.05).

It is possible that, with increasing breast fat, locally secreted leptin would exert most influence on the breast epithelial/stromal tissue, and plasma leptin would be less important. This could be a biological explanation for our finding that the mammographic density increased significantly across quartiles of plasma leptin levels in the medium stratum of breast fat, whereas this increase was not found in the upper stratum. If plasma leptin is associated with increased mammographic density in breasts only containing a modest amount of fat, however, we would expect the strongest association between plasma leptin concentration and mammographic density in the first stratum of breast fat. This is not what we found, indicating that our results may be due to chance.

There is some evidence for an association between estrogen and leptin, but the direction of this association is not clear. Estrogens have been shown by some investigators [58], but not all [59], to stimulate leptin production from female omental adipose tissue *in vitro*. Furthermore, it is unclear whether plasma leptin levels are affected by endogenous estrogens [60,61], by menopause or by the use of hormone replacement therapy [62]. In the present study, leptin levels did not differ with the use of postmenopausal hormone therapy, and neither was the association between plasma leptin concentration and mammographic density modified by hormone use (categorized as current use/non-current use; *P *for interaction = 0.23). We were unable to adjust for endogenous estrogen levels, but we adjusted for obesity, the major determinant of estrogen levels in postmenopausal women [63]. Leptin has been shown to stimulate the action of the aromatase enzyme in some women [64]. If estrogens mediate the effect of leptin on mammographic density, this would make estrogens an intermediate step in the association between leptin and mammographic density in our analyses, and should as such not be adjusted for.

The strengths of our study are the large sample size, and that it was a part of a population-based screening project with a high attendance rate. The reader of the mammograms was experienced, and was blinded to the characteristics of the women.

Limitations of our study include potential misclassification of plasma leptin levels because the participants were nonfasting [65] and their blood samples were not drawn at the same time of the day [66]. Some misclassification could arise because leptin was measured only once, although single measurements of circulating leptin appear to reflect long-term levels rather well [67,68]. These misclassifications would most probably bias our results towards the null. In the stratified analyses, the low number of women in some of the leptin quartiles limits the interpretation of our findings.

The BMI as a measure of body fat is not accurate [69], and there could be a residual confounding effect by body fat on the association between leptin concentration and mammographic density. This would tend to obscure a positive association. Furthermore, the estimation of breast fat using the nondense component on the mammogram, a two-dimensional picture, is a crude approximation of the volume of mammary adipose tissue. A study that estimated breast adipose tissue based on magnetic resonance imaging found that mammograms gave an especially poor estimate among women with dense breasts [70]. It is thus possible that we have misclassified the amount of fat among women with dense breasts, and we could therefore have some misclassification of the stratification variable.

Leptin was the only adipocytokine measured in the present study. However, several other fat-secreted substances have been suggested to provide links between obesity and breast cancer, such as adiponectin [71], hepatocyte growth factor [72] and IL-6 [7], perhaps through interfering with insulin resistance and estrogen synthesis [73]. The association of these substances with mammographic density is to our knowledge not known.

## Conclusion

Our results do not support a growth-stimulating effect of plasma leptin on mammographic density. Although weak and possibly due to chance, we found some evidence that breast fat may modify the association between the leptin concentration and mammographic density.

## Abbreviations

BMI = body mass index; *r*_sp _= Spearman's rank correlation.

## Competing interests

The authors declare that they have no competing interests.

## Authors' contributions

GU, JER and CAD discussed the design of the study. AS performed data analyses, and AS and GU drafted the manuscript. Classification of mammograms was performed by GU. JER carried out the immunoassays. ITG is the principal investigator of the Tromsø Mammography and Breast Cancer Study. YB was responsible for cleaning the data. MBV and GU contributed to the analysis and interpretation of the data, and MBV, JER, ITG, YB and CAD contributed to revisions of the manuscript. All authors approved the submitted manuscript.

## Figures and Tables

**Table 1:** Characteristics of women included in the study (*n *= 967)

Age (years)	60 (55–71)
Body mass index (kg/m^2^)	26.7 (13.1–47.2)
Number of full-term pregnancies	3 (0–11)
Age at first birth (*n *= 816)	22 (15–39)
Absolute mammographic density (cm^2^)	14.7 (0–155.2)
Percentage mammographic density (%)	9.6 (0–69.2)
Leptin (ng/ml)	14.5 (1.0–72.0)
Current postmenopausal hormone therapy use	253 (26)
Smoking (*n *= 906)	
Current daily smoking	268 (30)
Current nonsmoking	638 (70)

Data presented as the median (range) or number of observations (percentage).

**Table 2:** Selected variables in relation to leptin and absolute mammographic density^a^

	*n*	Leptin concentration (ng/ml)	Leptin concentration (ng/ml) adjusted for BMI^b^	*P *value^c^	Absolute mammographic density (cm^2^)	Absolute mammographic density (cm^2^) adjusted for BMI^b^	*P *value^c^
Age in tertiles (*n *= 967)							
55–58 years	318	14.0 (12.9–15.2)	13.4 (12.5–14.4)		13.3 (11.6–15.2)	12.6 (11.0–14.4)	
59–63 years	340	14.4 (13.3–15.6)	13.6 (12.7–14.6)		10.9 (9.6–12.4)	10.3 (9.0–11.7)	
64–71 years	309	14.1 (13.0–15.3)	13.4 (12.5–14.4)	0.98	10.5 (9.2–12.1)	10.1 (8.8–11.5)	0.01
Number of full-term pregnancies (*n *= 967)							
0	69	14.7 (12.4–17.5)	14.9 (12.9–17.2)		19.7 (15.0–26.1)	18.1 (13.9–23.7)	
1–2	385	12.9 (12.0–13.8)	12.6 (11.8–13.4)		13.8 (12.3–15.5)	13.0 (11.5–14.7)	
3	309	14.6 (13.5–15.9)	14.2 (13.2–15.3)		11.4 (10.0–13.0)	10.6 (9.3–12.19	
4 and more	204	15.9 (14.4–17.6)	13.8 (12.6–15.0)	0.30	6.8 (5.8–8.0)	6.8 (5.8–8.0)	<0.001
Age at first birth (*n *= 898) (82 have imputed values)							
< 20 years	129	16.3 (14.4–18.5)	14.4 (12.9–16.0)		7.2 (5.9–8.9)	7.1 (5.8–8.7)	
20–24 years	535	14.1 (13.2–15.0)	13.2 (12.5–14.0)		10.9 (9.9–12.1)	10.2 (9.2–11.4)	
>24 years	234	13.1 (12.0–14.4)	12.7 (11.7–14.0)	0.08	14.2 (12.1–16.5)	13.3 (11.4–15.5)	<0.001
Postmenopausal hormone use (*n *= 967)							
Current use	253	13.5 (12.3–14.8)	13.5 (12.5–14.6)		16.7 (14.4–19.3)	15.1 (13.0–17.6)	
Current nonuse	714	14.4 (13.6–15.2)	13.5 (12.8–14.2)	0.97	10.1 (9.2–11.0)	9.8 (8.9–10.7)	<0.001
Smoking (*n *= 906)							
Current nonsmoking	638	15.5 (14.7–16.4)	14.1 (13.4–14.8)		11.6 (10.6–12.8)	11.6 (10.5–12.8)	
Current daily smoking	268	11.4 (10.4–12.4)	12.4 (11.5–13.4)	0.01	11.2 (9.7–13.0)	9.4 (8.1–10.9)	0.02
Alcohol consumption (100 g alcohol/month) (*n *= 877)							
No alcohol	225	14.0 (12.7–15.4)	13.1 (12.1–14.2)		10.4 (8.9–12.2)	10.4 (8.9–12.1)	
Lower tertile (<0.42)	206	14.5 (13.1–16.0)	13.8 (13.0–15.1)		10.5 (8.9–12.4)	9.9 (8.4–11.7)	
Mid tertile (0.42–1.1)	213	15.1 (13.6–16.6)	13.9 (12.7–15.1)		10.5 (8.9–12.3)	10.2 (8.7–12.1)	
Upper tertile (>1.1)	233	13.5 (12.3–14.9)	13.7 (12.6–14.9)	0.48	14.2 (12.2–16.6)	12.7 (10.8–14.8)	0.06
BMI (*n *= 967)							
<25	330	9.0 (8.4–9.6)			16.4 (14.5–18.6)		
25–30	393	15.1 (14.2–16.0)			11.8 (10.5–13.3)		
>30	244	23.8 (22.0–25.8)		<0.001	6.8 (5.8–7.8)		<0.001
Waist circumference in tertiles (*n *= 965)							
<83.6 cm	323	9.1 (8.5–9.8)			16.3 (14.3–18.5)		
83.6–94.1 cm	326	14.4 (13.4–15.4)			12.7 (11.2–14.4)		
>94.1 cm	316	21.9 (20.4–23.5)		<0.001	7.3 (6.4–8.3)		<0.001
Mammographic nondense area in tertiles (*n *= 967)							
<120.1 cm^2^	325	9.8 (9.1–10.6)	12.4 (11.5–13.4)		20.3 (18.0–22.9)	18.2 (15.8–20.9)	
120.1–172.8 cm^2^	319	15.0 (14.0–16.2)	14.1 (13.1–15.2)		12.2 (10.8–13.8)	11.2 (9.8–12.9)	
>172.8 cm^2^	323	19.3 (17.9–20.8)	14.1 (13.1–15.2)	0.03	6.1 (5.4–6.9)	6.4 (5.6–7.5)	<0.001
Mammographic percentage density in tertiles (*n *= 967)							
< 4.5%	322	17.5 (16.2–19.0)	13.4 (12.5–14.4)				
4.5–15.5%	322	13.8 (12.7–14.9)	13.6 (12.7–14.6)				
>15.5%	323	11.8 (10.9–12.7)	13.5 (12.5–14.5)	0.9			

^a^Analysis of variance. Leptin and absolute mammographic density were log-transformed, reported means are back-transformed

^b^Adjusted for body mass index (BMI), categorized as <20, 20–22, 23–24, 25–26, 27–28, 29–31, 32–34, >34.

^c^For dichotomous variables, *P *values were obtained from analysis of variance, otherwise *P *values were obtained from test for trend. Analyses are adjusted for BMI; except for BMI and waist circumference, where the trend tests refer to the crude analyses.

**Table 3:** Unadjusted and adjusted means of mammographic density (95% confidence interval) in quartiles of plasma leptin concentration^a ^(*n *= 967)

	Quartile of plasma leptin concentration				*P*_trend_
		
	<9.1 ng/ml	9.1–14.5 ng/ml	14.6–24.0 ng/ml	>24.0 ng/ml	
*n*	251	234	243	239	
Absolute mammographic density (cm^2^)					
Unadjusted	13.8 (11.9–16.0)	13.5 (11.6–15.8)	9.4 (8.1–11.0)	9.9 (8.5–11.5)	<0.001
Adjusted^b^	14.7 (12.4–17.4)	14.9 (12.5–17.6)	10.7 (9.1–12.6)	11.2 (9.5–13.2)	0.001
Adjusted^b^, additionally adjusted for nondense area^c^	11.7 (9.9–13.7)	13.3 (11.3–15.7)	11.0 (9.5–12.9)	12.9 (11.1–15.1)	0.70
Adjusted^b^, additionally adjusted for BMI^d^	11.2 (9.4–13.3)	12.4 (10.4–14.9)	10.4 (8.8–12.3)	13.4 (11.3–15.9)	0.32
Adjusted^b^, additionally adjusted for nondense area^c ^and BMI^d^	10.7 (9.0–12.7)	12.2 (10.2–14.5)	10.4 (8.9–12.3)	13.3 (11.3–15.7)	0.16
Percent mammographic density (%)					
Unadjusted	11.3 (9.8–12.9)	9.6 (8.3–11.0)	6.4 (5.5–7.3)	6.2 (5.4–7.2)	<0.001
Adjusted^b^, and additionally adjusted for BMI^d^	8.6 (7.3–10.0)	9.1(7.7–10.6)	7.6 (6.5–8.8)	9.7 (8.3–11.2)	0.58

^a^Multiple linear regression. The absolute and percentage mammographic densities were log-transformed, reported means are back-transformed.

^b^Adjusted for age (tertiles), full-term pregnancies (none, 1–2, 3, >3), age at first birth (<20 years, 20–24 years, >24 years) and postmenopausal hormone therapy (current use/noncurrent use).

^c^Nondense mammographic area (tertiles).

^d^Body mass index (BMI), <20, 20–22, 23–24, 25–26, 27–28, 29–31, 32–34, >34.

**Table 4:** Adjusted means of absolute mammographic density^a ^(95% confidence interval) in quartiles of plasma leptin concentration, stratified by tertiles of nondense mammographic area (*n *= 967)

Nondense mammographic area	Quartile of plasma leptin concentration				*P*_trend_
		
	<9.1 ng/ml	9.1–14.5 ng/ml	14.6–24.0 ng/ml	>24.0 ng/ml	
First tertile (*n*)	140	90	55	40	
Mammographic density, adjusted^b^	19.1(16.2–22.6)	17.8 (14.7–21.5)	16.8 (13.5–20.9)	19.4 (15.0–25.2)	0.7
Mammographic density, adjusted^b^, additionally adjusted for BMI^c^	22.1 (18.2–26.8)	19.0 (15.6–23.1)	17.5 (14.0–21.9)	18.7 (14.4–24.3)	0.12
Second tertile (*n*)	68	82	94	75	
Mammographic density, adjusted^b^	10.6 (7.8–14.5)	14.1(10.4–19.1)	13.2 (10.1–17.4)	17.3 (13.1–22.8)	0.02
Mammographic density, adjusted^b^, additionally adjusted for BMI^c^	9.9 (7.2–13.6)	14.0 (10.3–19.0)	13.7 (10.3–18.2)	19.6 (14.6–26.3)	0.003
Third tertile (*n*)	43	62	94	124	
Mammographic density, adjusted^b^	7.5 (4.9–11.6)	10.4 (7.2–15.0)	6.2 (4.5–8.5)	7.0 (5.2–9.4)	0.22
Mammographic density, adjusted^b^, additionally adjusted for BMI^c^	8.2 (5.1–13.1)	11.3 (7.7–16.7)	7.5 (5.1–10.9)	8.6 (5.9–12.5)	0.63

^a^Multiple linear regression. Absolute mammographic density was log-transformed, reported means are back-transformed.

^b^Adjusted for age (tertiles), full-term pregnancies (none, 1–2, 3, >3), age at first birth (<20 years, 20–24 years, >24 years) and postmenopausal hormone therapy (current use/noncurrent use).

^c^Body mass index (BMI), <23, 23–24, 25–26, 27–28, >28.

## References

[B1] van den Brandt PA, Spiegelman D, Yaun SS, Adami HO, Beeson L, Folsom AR, Fraser G, Goldbohm RA, Graham S, Kushi L (2000). Pooled analysis of prospective cohort studies on height, weight, and breast cancer risk. Am J Epidemiol.

[B2] Key TJ, Appleby PN, Reeves GK, Roddam A, Dorgan JF, Longcope C, Stanczyk FZ, Stephenson HE, Falk RT, Miller R (2003). Body mass index, serum sex hormones, and breast cancer risk in postmenopausal women. J Natl Cancer Inst.

[B3] Lahmann PH, Hoffmann K, Allen N, Van Gils CH, Khaw KT, Tehard B, Berrino F, Tjonneland A, Bigaard J, Olsen A (2004). Body size and breast cancer risk: findings from the European prospective investigation into cancer and nutrition (EPIC). Int J Cancer.

[B4] Purohit A, Reed MJ (2002). Regulation of estrogen synthesis in postmenopausal women. Steroids.

[B5] Boyd NF, Stone J, Martin LJ, Jong R, Fishell E, Yaffe M, Hammond G, Minkin S (2002). The association of breast mitogens with mammographic densities. Br J Cancer.

[B6] Ahima RS, Flier JS (2000). Adipose tissue as an endocrine organ. Trends Endocrinol Metab.

[B7] Rose DP, Komninou D, Stephenson GD (2004). Obesity, adipocytokines, and insulin resistance in breast cancer. Obes Rev.

[B8] van Rossum EF, Nicklas BJ, Dennis KE, Berman DM, Goldberg AP (2000). Leptin responses to weight loss in postmenopausal women: relationship to sex-hormone binding globulin and visceral obesity. Obes Res.

[B9] Van Harmelen V, Reynisdottir S, Eriksson P, Thorne A, Hoffstedt J, Lonnqvist F, Arner P (1998). Leptin secretion from subcutaneous and visceral adipose tissue in women. Diabetes.

[B10] Bado A, Levasseur S, Attoub S, Kermorgant S, Laigneau JP, Bortoluzzi MN, Moizo L, Lehy T, Guerre-Millo M, Marchand-Brustel Y (1998). The stomach is a source of leptin. Nature.

[B11] Masuzaki H, Ogawa Y, Sagawa N, Hosoda K, Matsumoto T, Mise H, Nishimura H, Yoshimasa Y, Tanaka I, Mori T (1997). Nonadipose tissue production of leptin: leptin as a novel placenta-derived hormone in humans. Nat Med.

[B12] Reseland JE, Syversen U, Bakke I, Qvigstad G, Eide LG, Hjertner O, Gordeladze JO, Drevon CA (2001). Leptin is expressed in and secreted from primary cultures of human osteoblasts and promotes bone mineralization. J Bone Miner Res.

[B13] Solberg R, Aas V, Thoresen GH, Kase ET, Drevon CA, Rustan AC, Reseland JE (2005). Leptin expression in human primary skeletal muscle cells is reduced during differentiation. J Cell Biochem.

[B14] Smith-Kirwin SM, O'Connor DM, De Johnston J, Lancey ED, Hassink SG, Funanage VL (1998). Leptin expression in human mammary epithelial cells and breast milk. J Clin Endocrinol Metab.

[B15] Friedman JM, Halaas JL (1998). Leptin and the regulation of body weight in mammals. Nature.

[B16] Ahima RS, Flier JS (2000). Leptin. Annu Rev Physiol.

[B17] Otero M, Lago R, Lago F, Casanueva FF, Dieguez C, Gomez-Reino JJ, Gualillo O (2005). Leptin, from fat to inflammation: old questions and new insights. FEBS Lett.

[B18] Markowska A, Malendowicz K, Drews K (2004). The role of leptin in breast cancer. Eur J Gynaecol Oncol.

[B19] Somasundar P, McFadden DW, Hileman SM, Vona-Davis L (2004). Leptin is a growth factor in cancer. J Surg Res.

[B20] Sierra-Honigmann MR, Nath AK, Murakami C, Garcia-Cardena G, Papapetropoulos A, Sessa WC, Madge LA, Schechner JS, Schwabb MB, Polverini PJ (1998). Biological action of leptin as an angiogenic factor. Science.

[B21] Bouloumie A, Drexler HC, Lafontan M, Busse R (1998). Leptin, the product of Ob gene, promotes angiogenesis. Circ Res.

[B22] Iversen PO, Drevon CA, Reseland JE (2002). Prevention of leptin binding to its receptor suppresses rat leukemic cell growth by inhibiting angiogenesis. Blood.

[B23] Hu X, Juneja SC, Maihle NJ, Cleary MP (2002). Leptin – a growth factor in normal and malignant breast cells and for normal mammary gland development. J Natl Cancer Inst.

[B24] Johns PC, Yaffe MJ (1987). X-ray characterisation of normal and neoplastic breast tissues. Phys Med Biol.

[B25] Boyd NF, Lockwood GA, Byng JW, Tritchler DL, Yaffe MJ (1998). Mammographic densities and breast cancer risk. Cancer Epidemiol Biomarkers Prev.

[B26] Byrne C, Schairer C, Wolfe J, Parekh N, Salane M, Brinton LA, Hoover R, Haile R (1995). Mammographic features and breast cancer risk: effects with time, age, and menopause status. J Natl Cancer Inst.

[B27] Ursin G, Ma H, Wu AH, Bernstein L, Salane M, Parisky YR, Astrahan M, Siozon CC, Pike MC (2003). Mammographic density and breast cancer in three ethnic groups. Cancer Epidemiol Biomarkers Prev.

[B28] Ursin G, Hovanessian-Larsen L, Parisky YR, Pike MC, Wu AH (2005). Greatly increased occurrence of breast cancers in areas of mammographically dense tissue. Breast Cancer Res.

[B29] Boyd NF, Lockwood GA, Martin LJ, Byng JW, Yaffe MJ, Tritchler DL (2001). Mammographic density as a marker of susceptibility to breast cancer: a hypothesis. IARC Sci Publ.

[B30] Gram IT, Bremnes Y, Ursin G, Maskarinec G, Bjurstam N, Lund E (2005). Percentage density, Wolfe's and Tabar's mammographic patterns: agreement and association with risk factors for breast cancer. Breast Cancer Res.

[B31] Ma Z, Gingerich RL, Santiago JV, Klein S, Smith CH, Landt M (1996). Radioimmunoassay of leptin in human plasma. Clin Chem.

[B32] Ursin G, Astrahan MA, Salane M, Parisky YR, Pearce JG, Daniels JR, Pike MC, Spicer DV (1998). The detection of changes in mammographic densities. Cancer Epidemiol Biomarkers Prev.

[B33] Willett W, Stampfer MJ (1986). Total energy intake: implications for epidemiologic analyses. Am J Epidemiol.

[B34] Petridou E, Papadiamantis Y, Markopoulos C, Spanos E, Dessypris N, Trichopoulos D (2000). Leptin and insulin growth factor I in relation to breast cancer (Greece). Cancer Causes Control.

[B35] Mantzoros CS, Bolhke K, Moschos S, Cramer DW (1999). Leptin in relation to carcinoma in situ of the breast: a study of pre-menopausal cases and controls. Int J Cancer.

[B36] Stattin P, Soderberg S, Biessy C, Lenner P, Hallmans G, Kaaks R, Olsson T (2004). Plasma leptin and breast cancer risk: a prospective study in northern sweden. Breast Cancer Res Treat.

[B37] Furberg AS, Jasienska G, Bjurstam N, Torjesen PA, Emaus A, Lipson SF, Ellison PT, Thune I (2005). Metabolic and hormonal profiles: HDL cholesterol as a plausible biomarker of breast cancer risk. The Norwegian EBBA Study. Cancer Epidemiol Biomarkers Prev.

[B38] Lam PB, Vacek PM, Geller BM, Muss HB (2000). The association of increased weight, body mass index, and tissue density with the risk of breast carcinoma in Vermont. Cancer.

[B39] Salminen TM, Saarenmaa IE, Heikkila MM, Hakama M (1999). Unfavourable change in mammographic patterns and the breast cancer risk factors. Breast Cancer Res Treat.

[B40] Gram IT, Funkhouser E, Tabar L (1997). Anthropometric indices in relation to mammographic patterns among peri-menopausal women. Int J Cancer.

[B41] Boyd NF, Rommens JM, Vogt K, Lee V, Hopper JL, Yaffe MJ, Paterson AD (2005). Mammographic breast density as an intermediate phenotype for breast cancer. Lancet Oncol.

[B42] Capuco AV, Smith JJ, Waldo DR, Rexroad CE (1995). Influence of prepubertal dietary regimen on mammary growth of Holstein heifers. J Dairy Sci.

[B43] Silva LF, VandeHaar MJ, Weber Nielsen MS, Smith GW (2002). Evidence for a local effect of leptin in bovine mammary gland. J Dairy Sci.

[B44] Maskarinec G, Meng L (2000). A case-control study of mammographic densities in Hawaii. Breast Cancer Res Treat.

[B45] Chen Z, Wu AH, Gauderman WJ, Bernstein L, Ma H, Pike MC, Ursin G (2004). Does mammographic density reflect ethnic differences in breast cancer incidence rates?. Am J Epidemiol.

[B46] Maskarinec G, Meng L (2001). An investigation of soy intake and mammographic characteristics in Hawaii. Breast Cancer Res.

[B47] Maskarinec G, Takata Y, Franke AA, Williams AE, Murphy SP (2004). A 2-year soy intervention in premenopausal women does not change mammographic densities. J Nutr.

[B48] Boyd NF, Greenberg C, Lockwood G, Little L, Martin L, Byng J, Yaffe M, Tritchler D (1997). Effects at two years of a low-fat, high-carbohydrate diet on radiologic features of the breast: results from a randomized trial. Canadian Diet and Breast Cancer Prevention Study Group. J Natl Cancer Inst.

[B49] O'Brien SN, Welter BH, Price TM (1999). Presence of leptin in breast cell lines and breast tumors. Biochem Biophys Res Commun.

[B50] Laud K, Gourdou I, Pessemesse L, Peyrat JP, Djiane J (2002). Identification of leptin receptors in human breast cancer: functional activity in the T47-D breast cancer cell line. Mol Cell Endocrinol.

[B51] Okumura M, Yamamoto M, Sakuma H, Kojima T, Maruyama T, Jamali M, Cooper D, Yasuda K (2002). Leptin and high glucose stimulate cell proliferation in MCF-7 human breast cancer cells: reciprocal involvement of PKC-α and PPAR expression. Biochim Biophys Acta.

[B52] Cleary MP, Phillips FC, Getzin SC, Jacobson TL, Jacobson MK, Christensen TA, Juneja SC, Grande JP, Maihle NJ (2003). Genetically obese MMTV-TGF-α/Lep(ob)Lep(ob) female mice do not develop mammary tumors. Breast Cancer Res Treat.

[B53] Cleary MP, Juneja SC, Phillips FC, Hu X, Grande JP, Maihle NJ (2004). Leptin receptor-deficient MMTV-TGF-α/Lepr(db)Lepr(db) female mice do not develop oncogene-induced mammary tumors. Exp Biol Med (Maywood).

[B54] Ishikawa M, Kitayama J, Nagawa H (2004). Enhanced expression of leptin and leptin receptor (OB-R) in human breast cancer. Clin Cancer Res.

[B55] Caldefie-Chezet F, Damez M, de Latour M, Konska G, Mishellani F, Fusillier C, Guerry M, Penault-Llorca F, Guillot J, Vasson MP (2005). Leptin: a proliferative factor for breast cancer? Study on human ductal carcinoma. Biochem Biophys Res Commun.

[B56] Glasow A, Kiess W, Anderegg U, Berthold A, Bottner A, Kratzsch J (2001). Expression of leptin (Ob) and leptin receptor (Ob-R) in human fibroblasts: regulation of leptin secretion by insulin. J Clin Endocrinol Metab.

[B57] Alowami S, Troup S, Al Haddad S, Kirkpatrick I, Watson PH (2003). Mammographic density is related to stroma and stromal proteoglycan expression. Breast Cancer Res.

[B58] Casabiell X, Pineiro V, Peino R, Lage M, Camina J, Gallego R, Vallejo LG, Dieguez C, Casanueva FF (1998). Gender differences in both spontaneous and stimulated leptin secretion by human omental adipose tissue in vitro: dexamethasone and estradiol stimulate leptin release in women, but not in men. J Clin Endocrinol Metab.

[B59] Kristensen K, Pedersen SB, Richelsen B (2000). Interactions between sex steroid hormones and leptin in women. Studies in vivo and in vitro. Int J Obes Relat Metab Disord.

[B60] Thomas T, Burguera B, Melton LJ, Atkinson EJ, O'Fallon WM, Riggs BL, Khosla S (2000). Relationship of serum leptin levels with body composition and sex steroid and insulin levels in men and women. Metabolism.

[B61] Castracane VD, Kraemer GR, Ogden BW, Kraemer RR (2006). Interrelationships of serum estradiol, estrone, and estrone sulfate, adiposity, biochemical bone markers, and leptin in post-menopausal women. Maturitas.

[B62] Di Carlo C, Tommaselli GA, Nappi C (2002). Effects of sex steroid hormones and menopause on serum leptin concentrations. Gynecol Endocrinol.

[B63] Cauley JA, Gutai JP, Kuller LH, LeDonne D, Powell JG (1989). The epidemiology of serum sex hormones in postmenopausal women. Am J Epidemiol.

[B64] Magoffin DA, Weitsman SR, Aagarwal SK, Jakimiuk AJ (1999). Leptin regulation of aromatase activity in adipose stromal cells from regularly cycling women. Ginekol Pol.

[B65] Fogteloo AJ, Pijl H, Roelfsema F, Frolich M, Meinders AE (2004). Impact of meal timing and frequency on the twenty-four-hour leptin rhythm. Horm Res.

[B66] Licinio J, Negrao AB, Mantzoros C, Kaklamani V, Wong ML, Bongiorno PB, Negro PP, Mulla A, Veldhuis JD, Cearnal L (1998). Sex differences in circulating human leptin pulse amplitude: clinical implications. J Clin Endocrinol Metab.

[B67] Widjaja A, Levy JC, Morris RJ, Frayn KN, Humphreys SM, Horn R, von zur MA, Turner RC, Brabant G (2000). Determinants of within-subject variation of fasting serum leptin concentrations in healthy subjects. Exp Clin Endocrinol Diabetes.

[B68] Chu NF, Spiegelman D, Hotamisligil GS, Rifai N, Stampfer M, Rimm EB (2001). Plasma insulin, leptin, and soluble TNF receptors levels in relation to obesity-related atherogenic and thrombogenic cardiovascular disease risk factors among men. Atherosclerosis.

[B69] Fors H, Matsuoka H, Bosaeus I, Rosberg S, Wikland KA, Bjarnason R (1999). Serum leptin levels correlate with growth hormone secretion and body fat in children. J Clin Endocrinol Metab.

[B70] Lee NA, Rusinek H, Weinreb J, Chandra R, Toth H, Singer C, Newstead G (1997). Fatty and fibroglandular tissue volumes in the breasts of women 20–83 years old: comparison of X-ray mammography and computer-assisted MR imaging. AJR.

[B71] Mantzoros C, Petridou E, Dessypris N, Chavelas C, Dalamaga M, Alexe DM, Papadiamantis Y, Markopoulos C, Spanos E, Chrousos G (2004). Adiponectin and breast cancer risk. J Clin Endocrinol Metab.

[B72] Rahimi N, Saulnier R, Nakamura T, Park M, Elliott B (1994). Role of hepatocyte growth factor in breast cancer: a novel mitogenic factor secreted by adipocytes. DNA Cell Biol.

[B73] Lorincz AM, Sukumar S (2006). Molecular links between obesity and breast cancer. Endocr Relat Cancer.

